# Integrated interdisciplinary workflows for research on historical newspapers: Perspectives from humanities scholars, computer scientists, and librarians

**DOI:** 10.1002/asi.24565

**Published:** 2021-08-18

**Authors:** Sarah Oberbichler, Emanuela Boroş, Antoine Doucet, Jani Marjanen, Eva Pfanzelter, Juha Rautiainen, Hannu Toivonen, Mikko Tolonen

**Affiliations:** ^1^ Institute of Contemporary History University of Innsbruck Innsbruck Austria; ^2^ L3i Laboratory University of La Rochelle La Rochelle France; ^3^ Department of Digital Humanities University of Helsinki Helsinki Finland; ^4^ National Library of Finland Mikkeli Finland; ^5^ Department of Computer Science University of Helsinki Helsinki Finland

## Abstract

This article considers the interdisciplinary opportunities and challenges of working with digital cultural heritage, such as digitized historical newspapers, and proposes an integrated digital hermeneutics workflow to combine purely disciplinary research approaches from computer science, humanities, and library work. Common interests and motivations of the above‐mentioned disciplines have resulted in interdisciplinary projects and collaborations such as the NewsEye project, which is working on novel solutions on how digital heritage data is (re)searched, accessed, used, and analyzed. We argue that collaborations of different disciplines can benefit from a good understanding of the workflows and traditions of each of the disciplines involved but must find integrated approaches to successfully exploit the full potential of digitized sources. The paper is furthermore providing an insight into digital tools, methods, and hermeneutics in action, showing that integrated interdisciplinary research needs to build something in between the disciplines while respecting and understanding each other's expertise and expectations.

## 1 INTRODUCTION

Never before have collections of historical newspapers been so accessible to the public, a result preceded by a digitization process that has been ongoing for decades. Already in the 1990s, regional and national libraries have engaged and invested heavily in newspaper digitization (Terras, 2011), resulting in 17% of newspapers in European libraries being digitized in 2012 (Gooding, 2018). With the Europeana newspapers thematic collection, there is now a pan‐European newspaper portal available, giving access to 18 million newspaper pages of which 10 million pages were converted to full text.^1^ As this process of digitization continues, increasingly advanced techniques from the field of natural language processing (NLP) promise to optimize the historians' access to full‐text archives (Ehrmann et al., 2019). For the promise to come true, disciplinary research approaches from libraries, humanities, and computer science need to be replaced by interdisciplinary ones.

For the libraries, these developments are a significant advantage for reaching their core missions: preserving the originals, providing access services, and striving for the best possible interaction between users, information, and knowledge (Van den Bosch et al., 2009; Zhang et al., 2015). Libraries benefit from digitization because they can reach out to a potentially international and diverse audience (Nauta et al., 2017; Neudecker & Antonacopoulos, 2016). At the same time, the digitization efforts of libraries also benefit humanities scholars. Before digitization, working with newspapers was more daunting than inviting. The amount of material seemed insurmountable and the time that had to be invested to find information often stood in no relation to the actual value added to research (Abel, 2013).

With the advance of digitization and with more elaborate algorithms, tools, and methods developed by computer scientists, digital approaches have started to shape humanities' and especially historians' research, whose goal it is to study the past by exploring, analyzing, interpreting, and contextualizing primary sources such as newspaper articles (Korkeamäki & Kumpulainen, 2019; Oberbichler et al., 2020). Some historians need to create collections for further qualitative analysis (e.g., Gabrielatos, 2007) or use digital tools to support their qualitative research questions (e.g., Brait, 2020; Oberbichler, 2020a; Pfanzelter, 2020), some use text mining methods to identify linguistic patterns (Marjanen, Kurunmäki, et al., 2020), and others again study geographically distributed phenomena (Borruso, 2008).

In order to work with digital primary sources, according to Milligan (2019), historians need to gain new skills, especially in the practice of (digital) hermeneutics, which refers to the interpretation and understanding of large, digitized, or digitally born data sources. Fickers and van der Heijden (2020) defined digital hermeneutics as the “critical and self‐reflexive use of digital tools and technologies for the development of new research questions, the testing of analytical assumptions, and the production of sophisticated scientific interpretations.” According to Fickers (2020), digital hermeneutics combines critical reflection on historical practice as well as the training of practical “skills” in the sense of “digital literacy.” Moreover, digital source criticism as part of digital hermeneutics addresses both archival and historiographical issues “raised by changing logics of storage, new heuristics of retrieval, and methods of analysis and interpretation of digitized data.” This means that also the transformation from “sources” to “documents” to “data” for their epistemological implications needs to be questioned (Fickers, 2020). Therefore, there are two aspects to digital hermeneutics. On one hand, it is about the understanding of the data sources as digital objects. On the other hand, it is about the use of digital methodologies to process, handle, analyze, and interpret these data (Tolonen & Lahti, 2018). Although these aspects cannot be fully separated, here we focus on the latter perspective.

The growing need for digital tools and methods in the work of historians has made historical newspapers an interesting field of study for computer scientists, too. It provides opportunities for applying methods from NLP and brings up new challenges and questions. One of these is the analysis of text collections that are very noisy, due to imperfect output from optical character recognition (OCR) and because layout/segmentation processes produce imperfect results (Boroş, Hamdi, et al., 2020; Huynh et al., 2020; Nguyen, Jatowt, et al., 2020). Computer scientists can also address the complex issues of analyzing linguistic changes and variations over time or help grouping articles that address the same topic to support interpretations from different viewpoints and the extraction of events (Zosa, Hengchen, et al., 2020). The mission for computer scientists, in the context of historical newspapers, is to identify and formulate computational problems motivated by practices of historical research or emerging from the opportunities provided by digital newspaper corpora and to produce solutions (algorithms, computational models, software tools, etc.), in order to advance understanding of both computational problems and historical questions.

Due to these various needs, motivations, and interests, there is an increasing number of digital newspaper research projects that involve curators, geographers, computer scientists, and humanities researchers (e.g., NewsEye,^2^ ViralTexts,^3^ Oceanic Exchanges,^4^ impresso,^5^ Arkindex,^6^ and Living with Machines^7^). These projects are working toward collaborative and integrative approaches to get closer to the shared vision of “finding meaning” in digitized historical newspaper data. The Atlas of Digitized Newspapers (Beals & Bell, 2020), an open access guide prepared by leading computational periodicals researchers from six European countries, already made an important step toward facilitating more historically informed understandings of digitized newspapers for researchers across disciplines.

Integrated interdisciplinary research tries to build something in between the disciplines so they share more than just the problem. This differs from multidisciplinary collaboration, which generally means that people from different scientific fields come together, collaborate, and study a common question or problem with the goal of reaching common conclusions (Van den Besselaar & Heimeriks, 2001). Integrated interdisciplinary research requires going deeper than just saying something about a phenomenon from different perspectives (Ros & Oberbichler, 2020). This can be very rewarding, as the fields can fuel progress among themselves if aligned goals can be identified. This also includes the understanding of how each field works and which approaches are used for problem‐solving (Gooding, 2020). Integrated interdisciplinary work should bridge while at the same time define boundaries for each discipline.

In this paper, we use the concept of workflow to discuss integrated interdisciplinary collaboration. We argue that interdisciplinary teams can benefit from an understanding of the workflows and traditions of each of the disciplines involved and provide a schematic synthesis of typical workflow viewpoints of the disciplines, highlighting their similarities and differences. Integrated approaches are needed to successfully exploit the full potential of digitized sources. Therefore, we elaborate on research steps and tasks where all fields come together and eventually propose an integrated digital hermeneutics workflow for research work on digital cultural heritage.

## 2 MOTIVATION AND METHODOLOGY

The aim of this paper is to shed light on integrated interdisciplinary research between the curator, the humanities researcher, and the computer scientist, motivated by their shared vision of finding meaning in data. Much has been written about research with digital cultural heritage from a (digital) humanities scholars' perspective (e.g., Allen & Sieczkiewicz, 2010; Korkeamäki & Kumpulainen, 2019; Milligan, 2019), a (digital) librarian's perspective (e.g., Caffo, 2014; Millson‐Martula & Gunn, 2017), or a computer scientist's perspective (e.g., Koolen et al., 2009; Van den Bosch et al., 2009) separately. Some papers also bring together the viewpoints of at least two of the disciplines involved. Gooding (2020), Robinson et al. (2015), Zhang et al. (2015), and Angelaki et al. (2019), for example, address the relationship between the library and information science and digital as well as computational humanities, while the gaps between computer science and digital humanities are discussed, for example, by Biemann et al. (2014) and Crum et al. (2019). Kemman (2019) reflects on “digital humanities collaboration” and concludes that this collaboration seems to be “biased toward the humanities rather than balancing the digital and the humanities.” Kemman also points out that his quantitative approach (questionnaires) to investigate collaboration does not provide in‐depth insights into the development of common ground between researchers of different disciplines.

The present paper provides in‐depth insights by bringing together researchers from all three disciplines and opens up a broader discussion on shared as well as separated visions and motivations. The authors of this paper collaborate in the interdisciplinary and international NewsEye project (Doucet et al., 2020) from 2018 to 2021. In this project, computer scientists, mathematicians, historians, linguists, and librarians worked together on the development of new methods and tools for effective exploration and exploitation of historical newspapers. Regular meetings (online and in person), joint research and publications, extensive tool‐testing sessions, and internal as well as external workshops formed the basis for communication and the development of common ground. In this paper, we reflect on our experiences from collaboration and interdisciplinarity (collaborative auto‐ethnography [CAE]) (Hernandez et al., 2017), and we distill those reflections into a suggestion for an integrated interdisciplinary workflow. The insights are based on literature as well as on experiences closely connected to the research we conducted.

## 3 DIGITAL CULTURAL HERITAGE AT THE INTERSECTION OF (DIGITAL) HUMANITIES, LIBRARY, AND COMPUTER SCIENCE

### 3.1 Needs and motivations

**FIGURE 1:**
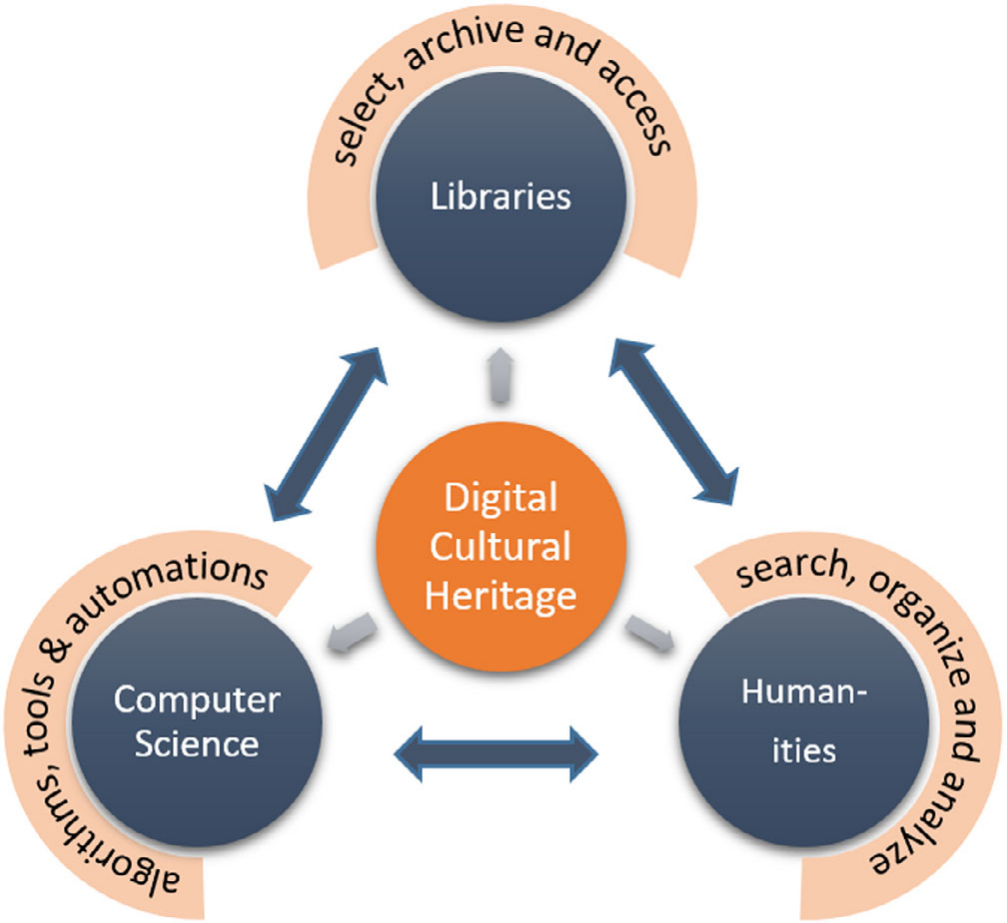
Tasks, interests, and interaction of computer science, library, and humanities when dealing with digital cultural heritage

Librarians, humanities scholars, and computer scientists are engaged in different aspects of digital cultural heritage such as digitized newspapers. Both librarians and humanities researchers share a common interest in collecting, organizing, and preserving digital sources, while computer scientists and humanities scholars share the interest in (methods for) accessing and discovering information and knowledge. Schematically presented, libraries are the providers of data and of efficient archiving and access services with the goal of reaching different users, while computer scientists could be described as producers and identifiers of solutions, algorithms, and tools (Figure 1). Humanities researchers, finally, are users of both technical solutions produced by computer scientists and digital material provided by libraries. All fields identify new problems and produce new solutions for progress in dealing with the digital cultural heritage they influence each other.

**FIGURE 2:**
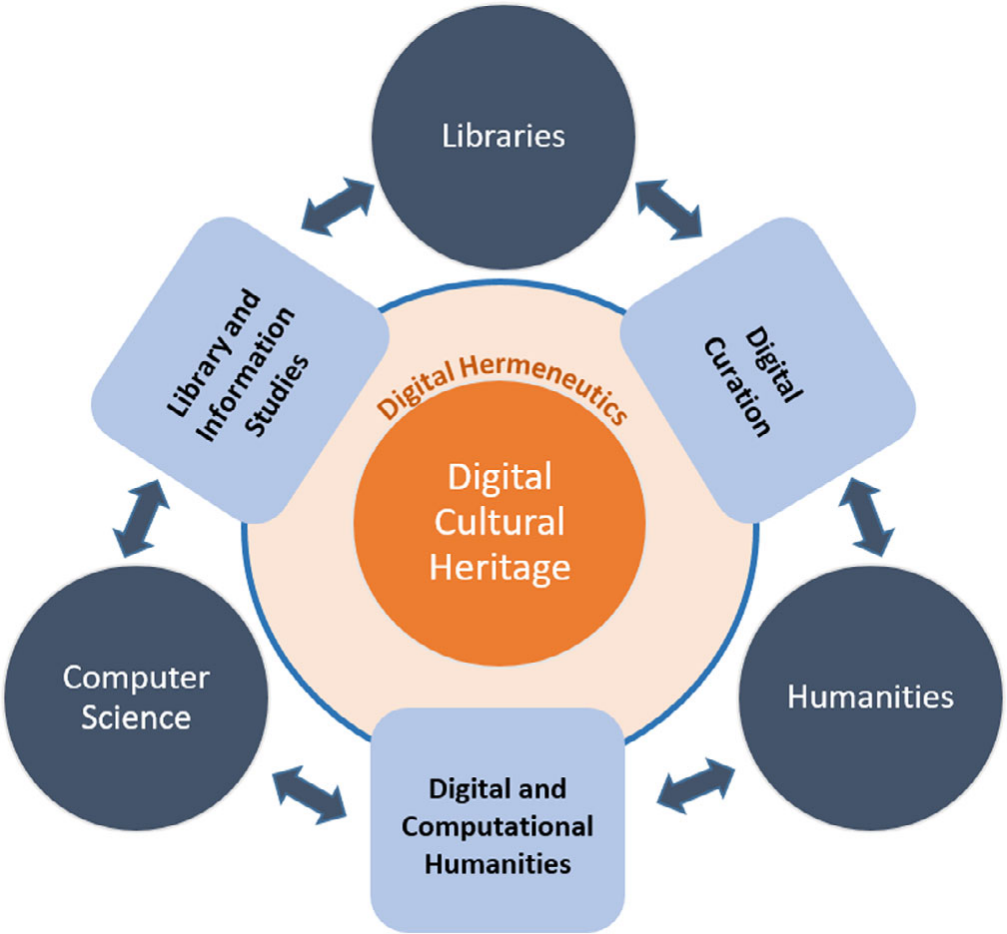
Interdisciplinary collaboration when working with digital cultural heritage

The development of interdisciplinary research fields such as digital or computational humanities, library, and information studies or digital curation as hybrid domains that bridge interdisciplinary boundaries as well as “traditional barriers between theory and practice, technological implementation, and scholarly reflection” (Flanders et al., 2007) is, considering the common needs and motivations, a logical consequence. These fields are again strongly interwoven (Figure 2).

Digital curation involves the preservation, promotion, and access to digital cultural heritage data as well as the migration of data into new formats, linking data or adding contextual information (Poole, 2013; Sabharwal, 2015). Library and information studies focus on the application of technical achievements to library operations and services (Hayes, 1985). Digital or computational humanities produce new methodological approaches (with emerging challenges such as multilingualism or multimedia; Liu, 2020) while critically reflecting on the practices related to digital technologies thus engaging in fruitful discussions on digital hermeneutics as an “art of in‐betweenness” (Fickers, 2019) requiring interdisciplinarity as well as new skills.

### 3.2 Differences and similarities

There are differences between the disciplines that can be difficult to bridge. For computer scientists in the project, it was most interesting to push the limits of methodological development, whereas humanities researchers were more prone to transfer established and traditional humanities' research methods to be used in the digital world. For librarians, the interests of the individual researchers or the project were sometimes secondary to what is most useful for a wider user group. For example, building individually tailored corpora for further qualitative analysis was an important research step for some humanities scholars but rather uninteresting for computer scientists and rarely considered by librarians. At the same time, humanities scholars sometimes found it difficult to integrate new developed methods in their research if those methods were “merely” of a quantitative nature or outside of their “research comfort zones.”

In order to carry out joint research and to move toward more integrated approaches, the project team had to find common ground for several challenges. Especially in the project's tool‐testing sessions, it became clear that humanities scholars often find functionalities provided by libraries or computer scientists rigid or bulky and sometimes disadvantageous for needed digital hermeneutics and digital source, tools and methods criticism (which in turn was sometimes deemed unnecessary for the other disciplines) (Pfanzelter et al., 2021). Humanities researchers and especially historians tend to see digital computational methods merely as a modernization of the traditional auxiliary sciences. For them, to be able to control each step in the research process applying traditional source (as well as tool, method, interface, etc.) criticism seems crucial and it explains why they are sometimes loath to trust the algorithms “to do their magic” (Korkeamäki & Kumpulainen, 2019).

While there is justification for not fully relying on computational tools, we also learned that these arguments are sometimes used to defend an unnecessarily conservative attitude that frowns upon new opportunities provided by large digital corpora and computational approaches. Many algorithms are actually transparent for the computationally literate (e.g., section 4.3), and humanities researchers do already trust the “magic” of many algorithms, for example, in optical character recognition (OCR), part‐of‐speech (POS) tagging, or named entity recognition (NER).

Computer scientists can be equally strict about their discipline showing a tendency to analyze and structure the tasks and problems of historians from their own viewpoint. They sometimes find it difficult to appreciate the concepts, methods, and actual needs of historians or libraries when the goals could not be aligned with the goals and practices of research in computer science. Further complications became visible when prototype implementations of a promising new algorithm needed to be put into practice by historians. Academic computer science researchers sometimes were unwilling to meet the demand for the extra engineering work that makes digital methods actually useful to humanities scholars. Likewise, we experienced that humanities scholars sometimes were unwilling to do the tedious work of manually compiling annotations to allow for the evaluation of computer science methods, and more critically for the training of machine learning models.

Libraries, on the other hand, have their own practices and standards that guide their work. These may be a hindrance for fruitful co‐operation if not understood and addressed properly. For example, machine readable cataloging (MARC) standards, first developed in the 1960s, are still widely used in libraries despite their known limitations in the modern computing environment (Park & Kipp, 2019; Tennant, 2004). Also, selection criteria of libraries for digitization (and of what is being made accessible to users) often do not align with the interests of users and researchers and the choice for specific periods and newspaper issues as well as incomplete digitization process affected the landscape of digitized newspapers, and sometimes also skewed chronological representativeness (Hauswedell et al., 2020). Finally, digital transformation of cultural heritage is no doubt also political (Thylstrup, 2019). Competing requirements and demands, such as copyright and technical feasibility, need to be balanced (IFLA, 2002).

### 3.3 A workflow‐oriented view to interdisciplinary collaboration

**FIGURE 3:**
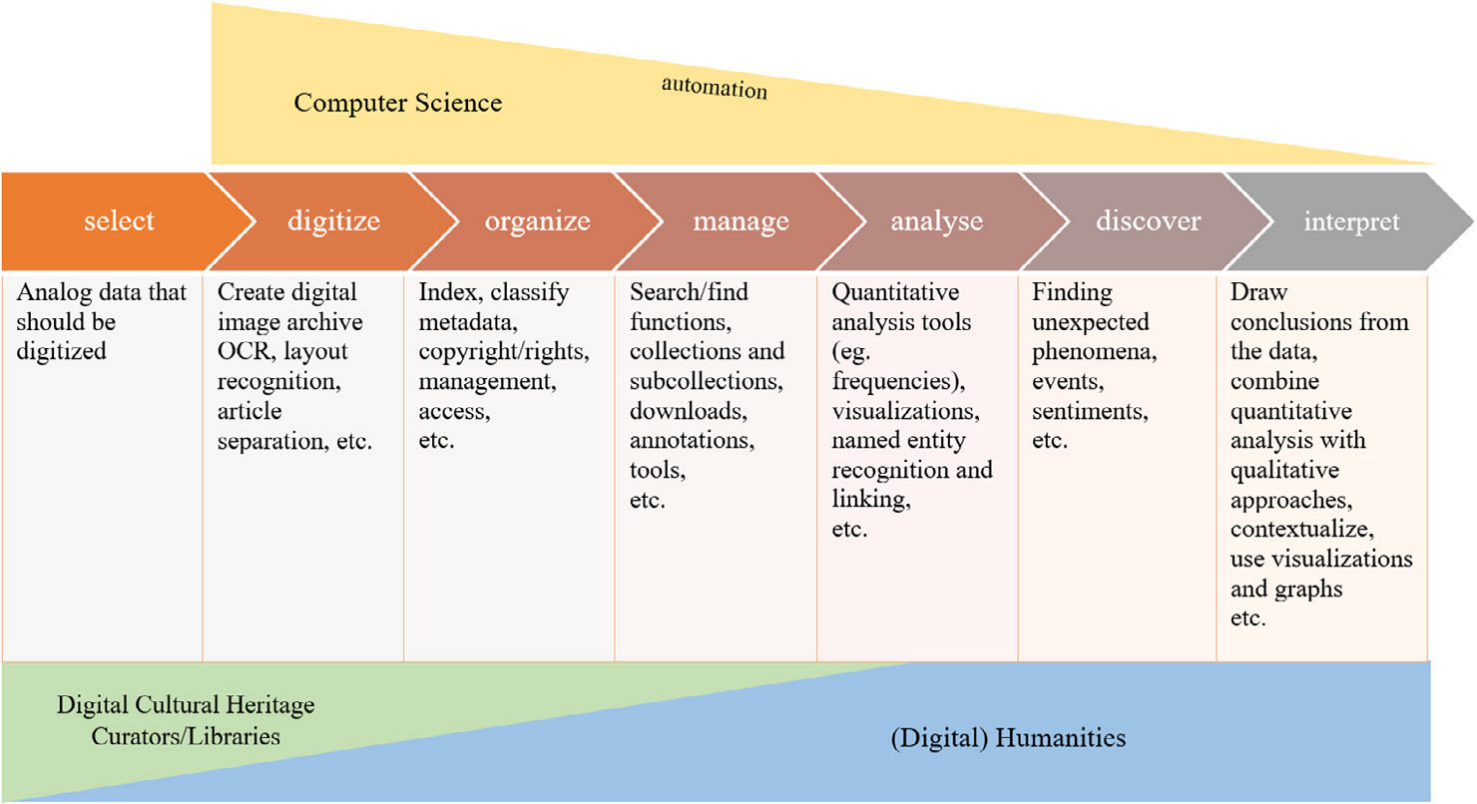
Involvement of each discipline and areas of application of (automated) computational methods

A main reason for the difficulty of finding common ground was found to be in different workflows, and especially different appreciations of the steps in the workflows. Even though all disciplines share the goal of understanding and developing novel solutions for the work with digital newspaper collections and engage in the identification of new problems, they differ in their respective research questions, objectives, and in the areas of application of computational methods (see Figure 3).

Not all areas where digital methods, tools, and algorithms are being applied involve all three disciplines but they have different needs and priorities. In addition, each field has different traditions and ideas of how digital methods are applied to digital cultural heritage.

While the tasks of digitization, structuring and management of cultural heritage materials are steps shared in all disciplines, for digital libraries, the digitization comes along with an evaluation process regarding ethical considerations. For example, if indexing is based only on the textual content of a source, it may be compromised by a lack of context and make it difficult for users to search (National Research Council (U.S.) & Committee on an Information Technology Strategy for the Library of Congress, 2000). For libraries—unlike computer scientists, providing context, for example, in the form of metadata is not just a technical but also an intellectual and labor‐intensive task.

For computer scientists, on the other hand, the involvement of digital methods in historical research has given rise to a structured pipeline view of subtasks of the work, which starts with digitization and ends with the interpretation. Software tools typically aim to address one or several tasks at a time, leading to workflows involving distinct steps. This is not only a technical coincidence or inconvenience but rather a reflection of the computer science approach: complex processes and problems are divided and structured into smaller ones so that each one can be analyzed, solved, and implemented as independently of others as possible. Minimal interaction and clear interfaces between the subtasks help manage the problem and software complexity. Unfortunately, a rigid division to disjoint subtasks and minimal interaction between them does not reflect the needs and processes of humanities researchers well.

Humanities researchers, again, are more interested in the tasks involving the organization, management, and analysis of the data and their highly individual access to the digital material and data is difficult to merge into standardized workflows or pipelines. They aim at discovering novel things in the data and interpreting their findings in creative ways. They tend to combine quantitative and qualitative methods (e.g., Berg, 2020), by going back and forth between the data, the metadata, and qualitative analysis (Oberbichler, 2020b) and by drawing conclusions combining all research steps. At the same time, qualitative analysis only plays a minimal role in the technical infrastructures provided by computer scientists and libraries. This results from the difficulty of automation in the latter tasks. While automation is at the heart of computer scientists' way of working, the challenges seem to become increasingly difficult and error‐prone with each step in the pipeline. If the *digitize*, *organize* and *manage* tasks from Figure 3 above, for example, have a success rate of 80 % each, an oversimplification is that after three steps the error rate cumulates and ends up with 51.2 % success rate (0.8 × 0.8 × 0.8). This means that the role of historians increases as less support is available via computational means.

Cultural heritage institutions are becoming increasingly aware of the different expectations of user groups and audiences. Digital labs in libraries are emerging as investigative areas that aim at helping different kinds of users to experiment with digital content “through competitions, awards, projects, exhibitions, and other engagement activities” (Chambers et al., 2019). The labs, but also the use of tools existing outside the library environment, could help to address growing user expectations toward text mining methods (Ehrmann et al., 2019) without complicating the uses of archival material within the libraries. The challenge here remains that tools developed in research projects are usually tested with small datasets as compared to digital collections in libraries—an issue that was also found to be true for the NewsEye team. For example, the dataset used in the competition of the International Conference on Document Analysis and Recognition (ICDAR) contained approximately 2,000 pages in 2017 (Chiron et al., 2017) and 15,221 pages in 2019 (Rigaud et al., 2019). For comparison, the present digitized newspaper collection of the National Library of Finland contains over 11 million newspaper pages and Austrian Newspapers Online collection ANNO contains over 23 million newspaper pages. In the NewsEye project, the dataset consists of 1.5 million newspaper pages. This is a fraction of the digitized collections of the participating libraries, but it should be sufficient to provide experience with the scalability of the tools used and developed in the project.

### 3.4 Creating an integrated interdisciplinary workflow

In order to provide a process for successful collaboration and communication, the differences and commonalities between disciplines need to be considered. Merging of applications, tasks, and traditions, involving mixed‐method approaches as well as increased interaction between the disciplines, has been identified as a possible common objective (Mäkelä et al., 2019). Based on our experience described above, we conclude that this involves openness for compromises: (digital) humanities researchers need to be open for analytical and process‐oriented thinking that takes the opportunities as well as limits of computational and digitization processes into account. Computer scientists need to be open to humanities' hermeneutic traditions and engage in the interpretative and contextualization processes of humanities research more deeply. Libraries need to be open for outside involvement: They could provide spaces for collaboration, engage in interdisciplinary projects when digitizing their archival collections, and help fund experiments.

**FIGURE 4:**
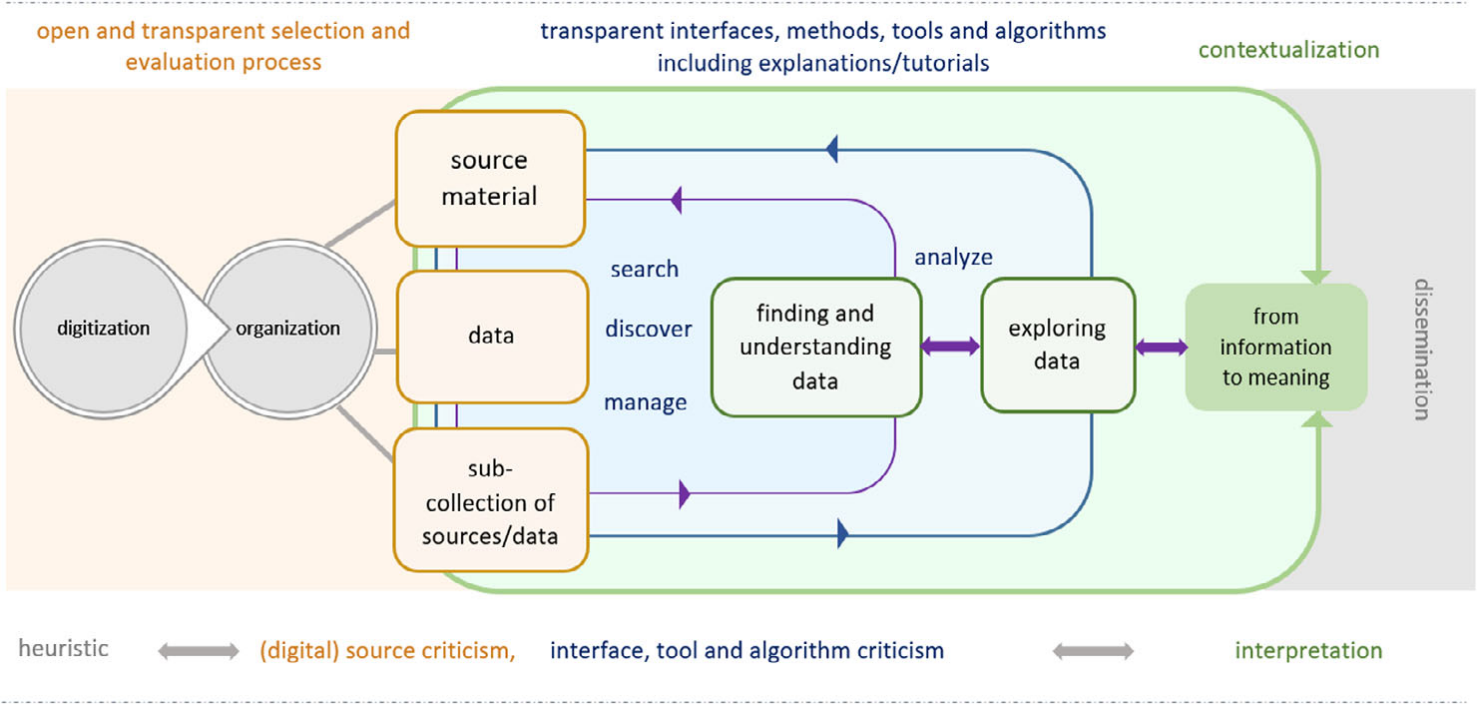
Interdisciplinary digital hermeneutics workflow

Digital hermeneutics provides a potential framework for creating an integrated workflow where these concerns can be brought into practice (Figure 4). Already according to Johann Gustav Droysen's historical method (1857/1882) (Leyh, 1977), historical knowledge is gained through three research steps: heuristics, (source) criticism, and interpretation. Owing to many methodological reiterations and a turn to hermeneutics (Gadamer, 1975), all three research steps are also relevant for digital environments. For historians, heuristics is seen as the step where researchers elaborate on their research questions and find, select as well as collect (primary) source material (Figure 4, left). In the second step, they critically engage with the selected material asking questions about relevancy, authenticity, appropriateness, materiality, and so on (Figure 4, middle). The third step consists of the interpretation of the material, the gaining of knowledge by skillfully combining information and data (Figure 4, right).

The integrated hermeneutics workflow of Figure 4 differs from the pipeline model (Figure 3) in three main aspects. First, it emphasizes the role of data (source material, data, subcollections). Second, it emphasizes the importance of iterative (qualitative) analytical steps over the data, in order to gradually gain deeper insights into it (finding and understanding data, exploring it, deriving meaning). Third, it emphasizes critical reflections of both data and tools in the spirit of hermeneutics, which requires openness and transparency of the methods and tools. These choices reflect the humanities researcher's view in contrast to the predominantly computational view in the pipeline model.

The first steps of the workflow (Figure 4, left) are essentially the same as above. Libraries as the curators of cultural heritage should be aware of the importance of why and how they select material for digitization, and they have to trace each step of these processes. The sharing of metadata—information that describes a physical or electronic resource (Riley, 2017)—is crucial in this context as well (Lahti et al., 2019; Mäkelä et al., 2019; Tolonen et al., 2017, 2018).

The middle and right parts of the integrated workflow present additional challenges to the computer scientists. Open and transparent selection and evaluation processes, as well as transparent interfaces, methods, tools, and algorithms, are needed to support the critical reflection of humanities scholars in the process of searching, discovering, managing, and analyzing digital cultural heritage materials, as well as in tool and algorithm criticism. Interaction and iteration between the analytical, exploratory, interpretative, and contextualizing steps implies tighter integration between them, making both system and interface design increasingly challenging.

The proposed interdisciplinary digital hermeneutics workflow is a concept rather than a concrete architecture. It sums up our experiences and insights regarding interdisciplinary research in a simple model. While this workflow model was developed at the end of the NewsEye project, ideally, such a workflow should be designed at the beginning of an interdisciplinary project. We believe that the model of Figure 4 can be a useful starting point for planning interdisciplinary collaboration in future research projects on historical newspapers.

## 4 INTERDISCIPLINARY DIGITAL HERMENEUTICS IN ACTION: THREE EXAMPLES

The following sections show how the integrated digital hermeneutics workflow can support critical engagement with digitized source material.^8^ For simplicity, on the one hand, we focus on the high‐level workflow steps of heuristics, (source) criticism, and interpretation, and on the other hand give examples of technical improvements, new tools, and methods that can be used in the workflow for the source preparation, organization, management, and analysis.

### 4.1 Digitization (optical character recognition and layout analysis)

Source preparation—which contains all digitization steps including layout analysis and article separation—is among the first steps to provide digital access to cultural heritage material such as newspapers. The inherent processes are not neutral technically but influenced by subpolitical processes that accompany the selection for digitization in the first place (Thylstrup, 2019) and they are thus part of the integrated hermeneutics workflow (Figure 4), which also means that the selection and evaluation process should be made transparent to users.

From a technological perspective, the automated conversion of images of historical documents to electronic text via optical character recognition (OCR) or handwritten character recognition (HTR) is a first automated step in this workflow. Layout analysis and “article separation” dividing the OCRed text into news‐units, is a second essential step in this process. Some large corpora have long existed in digitized form, but one of the main challenges researchers face when using digitized historical material is the poor quality of the text automatically extracted by OCR systems (Boroş, Linhares Pontes, et al., 2020; Huynh et al., 2020; Miller et al., 2000; Mutuvi et al., 2018; Nguyen, Boroş, et al., 2020). This long‐known problem affects the research process from the beginning to the end. Digital newspaper collections offered by national and regional libraries are no exception. Due to the quality of the original paper versions and without adequate software or tools, digitization initiated before 2017 (or even later) often has unsatisfactory OCR quality, and sometimes there is little transparency relating to the process of digitization.

The awareness of the importance of metadata only grew over time. This concerns the analogue material, for example, the state of the paper versions, publication frequency, completeness of the digitized collection, political orientation, changing editorial offices, languages, and so on, but also the digitization processes, for example, utilized software and hardware, image resolution, text recognition accuracy, availability, ownership, and so on. Without this information digital source (and tools, methods, interface, etc.) criticism is difficult to perform and the reliability of digital data collections diminishes. Transparency on OCR issues and digitization processes like the overall quality of OCR (or HTR) helps researchers to better estimate the impact of OCR problems for further research.

But insufficient OCR, incomplete collections or unsatisfactory layout segmentation do not necessarily have to be accepted as an unchangeable fact. One of the options is to re‐OCR digitized documents if the quality of text recognition is not satisfactory (Kettunen et al., 2019; Neudecker et al., 2019). Transkribus^9^ is a comprehensive platform for the recognition and transcription of historical documents, widely used in this task. Within the NewsEye project, for example, a dataset of some 1.5 million pages from the participating national libraries of Austria, France, and Finland was re‐OCRed with Transkribus by the partners at the University of Innsbruck and the University of Rostock. The process led to impressive improvements in OCR, producing output with character error rates below 1 %.

Re‐OCRing documents is not always realistic for very large collections due to computational costs and, even more critically, due to low image resolution resulting from earlier digitization campaigns. Thus, another approach to improve text quality without re‐OCRing is to work with OCR postcorrection, for which NewsEye researchers have provided exhaustive studies (Nguyen et al., 2019) relying on deep learning and OCR error analysis, as well as public benchmarks (Chiron et al., 2017; Rigaud et al., 2019). In addition, computer scientists from the universities of La Rochelle and Helsinki are researching automated tools and methods that are able to remove residual OCR errors (also known as OCR noise), by either applying different spelling correction methods or using advanced neural methods along with word representations (Hämäläinen & Hengchen, 2019; Huynh et al., 2020).

In the NewsEye project, the re‐OCRed text was uploaded to the NewsEye platform,^10^ a prototype interface for digitized newspapers, that was created as an experimental research lab. With better OCR, it soon became evident that faulty automated layout analysis and article separation are an issue for the creation of subcollections. The latter were deemed especially important for researchers who engage in qualitative analysis, close and wide reading, and who want to be able to export entire article collections for further analysis. It is also an issue for automated text analysis methods that expect (reliably recognized) separate articles as input.

### 4.2 Search, organization, and management

For many historians, finding specific articles as well as creating subcollections is essential for their research (Pfanzelter et al., 2021). Researchers therefore need to be able to search, structure, and organize digitally available historical resources and adapt the collections to their needs: this builds the middle part of the interdisciplinary hermeneutics workflow of Figure 4. The tasks of searching for articles or creating subcorpora can range from identifying tangible keywords (e.g., people, places, events) to more abstract, varied, and subtler concepts (e.g., themes, topics, etc.) that could foster further research toward finding patterns regarding cultural changes, variations in gender bias across the historical periods, emerging technological trends, or transitions to new political ideas.

Case studies, as mentioned in Pfanzelter et al. (2021), have shown that keywords are still essential in finding articles that are relevant to answer individual research questions. At the same time, the keyword search as it can be found in many newspaper interfaces suffers from weaknesses, like alternative spellings, polysemy, abbreviations, changing word usage, idioms, misspellings, or omissions (Bair & Carlson, 2008). In addition, many search requests are difficult to define conceptually and hard, if not impossible, to trace by single keywords alone. At the same time, when building topic‐specific corpora using keyword queries, there is always a compromise between the precision and the recall of such search queries (Chowdhury, 2010; Gabrielatos, 2007). Methods to improve keyword searches as well as keyword criticism are therefore important in the process of finding and understanding data.

Most newspaper interfaces of large digitized newspaper archives allow an advanced keyword search with a range of search options (boolean operators, wildcards, phrase search, etc.), but they do not support finding new relevant keywords (keywords that are similar to the search keyword) and they do not allow tracking changing word usage or spelling mistakes. The NewsEye partners from the Universities of La Rochelle and Helsinki are developing tools to support a more fine‐grained keyword search with the help of word representations. Word representations or embeddings are based on the distribution of words. They are created by using large collections of texts and detect words that are semantically or syntactically similar to a given word. The suggestions help users to find words that are used in similar contexts as well as to recognize alternative spellings. For data providers it is important to note, that they also point to frequently occurring OCR errors (for further references, see Wevers & Koolen, 2020).

In order to meet the need of creating individual collections, interfaces like Media Monitoring of the Past^11^ or the NewsEye platform provide finer‐grained keywords. Dates, locations, person names, organizations, or events can be interesting for historians (Blanke et al., 2020; Sprugnoli, 2018). In NewsEye, such keywords are identified by multilingual named entity recognition (Boroş, Hamdi, et al., 2020) and linking (Boroş, Linhares Pontes, et al., 2020) as well as event detection (Nguyen, Boroş, et al., 2020). Current research is using such methods also for linking related documents across languages in a multilingual collection (Zosa, Granroth‐Wilding, et al., 2020).

Natural language processing methods can further support the classification of, for example, relevant and nonrelevant articles within a collection by taking the context of keywords (article in which a keyword appears) into account. Our experiments show that collections can be automatically grouped with statistical methods such as latent topic modeling (LDA) and distance measures as in the Jensen‐Shannon divergence (JSD) (Oberbichler, 2020c).

However, the preparation and preprocessing of digital newspaper collections require a fair amount of work as well as a willingness to experiment with text mining methods. In order to support a larger user group in these efforts, YouTube tutorials^12^ or ready‐made *Jupyter* notebooks, for example, can be a great help and be essential for interdisciplinary digital hermeneutic workflows, since they are transparent, allow for explanations and can be developed in collaboration. There are already a number of projects, such as the GLAM Workbench,^13^ the BVMC Labs,^14^ or NewsEye notebooks^15^ that provide code via notebooks for the use with different kinds of sources and for different approaches.

### 4.3 Analysis of language use patterns

Many historians use language data in newspapers as an entry point for studying historical processes that the newspapers reported on, but quite a few historians are also interested in studying the discourse in its own right, meaning that there is a renewed interest in language as an indicator of historical change. Such studies move from using interfaces and algorithmic methods to find relevant sources to using them to produce representations of changes in past discourses. For instance, a topic model can be used to cluster similar documents and produce a subcorpus for closer study, but it can also be used as an indicator for something that is studied in the data. Following Pääkkönen & Ylikoski (2020), the former can be called topic instrumentalism, whereas the latter is a form of topic realism. This distinction can also be used for a general approach to quantification in using large‐scale text datasets.

Language use patterns can be approached in very simple ways or using complex algorithmic methods of analysis. Simplest forms of quantifying such patterns consist of studying word frequencies (Church & Hanks, 1989), whereas more complex studies deploy corpus linguistic methods based on keyness, TF‐IDF (term frequency‐inverse document frequency), or computationally more complex methods like topic models (Wallach, 2006) or word embeddings (Mikolov et al., 2013). The way digital newspaper collections are available for research greatly affects how researchers can assess patterns. With data dumps of full texts (for methods to create digital collections as data set see Candela et al., 2020), the possibilities to quantitatively analyze and visualize frequency‐related issues are almost endless, but demand data handling skills and generally require collaboration between scholars with domain expertise and computer science know‐how. However, bespoken interfaces for particular newspaper collections generally provide some possibilities for researchers to evaluate language use patterns (Ehrmann et al., 2019; Pfanzelter et al., 2021). Nevertheless, reasons for changes in frequencies vary and making sense of them often requires possibilities to explore the frequencies from different perspectives.

Frequency analysis often falls between the cracks in the sense that it is methodologically unchallenging for computer scientists but can be difficult for humanities domain experts to master themselves. The tools provided in graphical user interfaces are often lacking in possibilities to normalize frequencies with respect to different baselines. For instance, comparing the occurrences of politically laden words in two newspapers is not very informative unless we can normalize them against how many tokens the newspapers published altogether or some other relevant baseline. Only that gives us comparable results for interpretation (see Jenset & McGillivray, 2017, pp. 1–35). Setting a relevant baseline can be a nontrivial task as the normalization may end up reflecting something else than what was originally intended (for instance, editorial interests rather than differences in word preferences). Hence, an integrated hermeneutics workflow requires expertise both by domain experts (what is relevant, what is already known) and computer scientists (what can be computed, what kind of metadata is available), while a critical reflection on frequency and text mining tools as well as on the historical practice itself is essential in the process of analyzing and interpreting data.

More elaborate methods for the analysis of historical change cannot be used without tailoring them to the specific humanities research questions. This requires going beyond the interface, regardless of whether complicated tools are incorporated in it or not, and demands collaboration between researchers with expertise in the humanities and computer science (Van Gorp et al., 2019). For instance, word embeddings can be used to suggest new keywords for exploration, but they are also used to trace changes in meaning over time, that is, answering questions traditionally associated with qualitative study in the history of ideas or historical semantics (Friedrich & Biemann, 2016; Wevers & Koolen, 2020). However, using these methods requires an understanding of the parameters used (such as frequency thresholds for word types), choosing clustering methods (such as k‐means or affinity propagation) or simply assessing how different algorithms (such Word2Vec or Scot) react to general word type frequency. Choices like these are not only issues for computation, but also have effects for humanities interpretation (Marjanen, Kurunmäki, et al., 2020).

Compared to word embeddings, the use of topic models to represent a historical change in language use patterns is even more difficult, as the immediate link between the topics produced by the model and language use recorded in the data is weaker (Marjanen, Zosa, et al., 2020). Still, topic models are currently most effective if we want to understand the totality of data and changes in the discursive landscape captured in that data. It is, however, an open question of how the different methods (such as time‐sliced LDA or dynamic topic modeling (DTM)) represent historical change (Zosa, Hengchen, et al., 2020). The evaluation of the quality of the representations is difficult, as objective ground truth for historical change in discourse cannot be produced, but an assessment still needs to be made to support the humanities' interpretation. This, again, requires a dialogue between the humanities and computer science.

## 5 DISCUSSION

Interdisciplinary collaboration can be difficult even when all participants have good intentions. Researchers from all disciplines within the NewsEye project are bonded by a shared interest in digital historical newspapers and a willingness to engage in collaborative research and method development. While common interests and goals prevailed, unmet expectations or failed collaborations also led to occasional withdrawal into one's own discipline. To overcome the differences, the team needed to start thinking about the roots for these issues and consequently about possible solutions. The three examples in the previous section highlight some of these aspects.

We developed a workflow view to our project, and eventually a digital hermeneutics workflow as a model for integrated interdisciplinary research on historical newspapers. While it is clear that a single model cannot fully cover the needs and workflows of computer scientists, humanities scholars, and librarians, we believe that the workflow viewpoint can help integrate digital interfaces, tools, methods, and algorithms in digital hermeneutics, as was illustrated with the three examples. The multipart and interdisciplinary workflow proposed in this paper is intended to serve as an example and a reference point.

In order to reach integrated digital hermeneutics, we learned the importance of (a) grasping the difference between multidisciplinary and interdisciplinary research, (b) understanding each discipline's motivations, habits and expectations toward each other, and (c) accepting that every discipline needs to step up and make an effort for the other ones to be able to do actual research. We briefly discuss how the workflow view contributes toward these points.

First, designing a workflow for one's own project—or adapting the one from this paper—forces the researchers to explicitly consider how integrated and interdisciplinary they want to be, or if their project is more about multidisciplinary collaboration.

Second, mutual understanding between disciplines emerges from communication. Reflecting on and talking about workflows is one of the first steps to find a shared view of each other's perspectives, habits, and traditions. Communication about the expectations and limits of each discipline seems essential, as implicit assumptions about them can often be wrong. This requires investing time and effort to understand each discipline.

Third, a detailed workflow—more detailed than what can be presented here—makes the necessary tasks and components explicit. This helps identify practical boundaries of each discipline and negotiate compromises between them, for example, agreeing where different parties contribute to essential tasks outside their interests but within their expertise.

For instance, an observation in the NewsEye project, unexpected to computer science researchers, was the critical need for (digital) humanities scholars to be able to define subcollections, to be saved and exported, because these offer better opportunities for them to do research beyond quantitative big‐data evaluations. While this seemed a theoretically trivial matter to computer science researchers, it is now one of the most used features of the NewsEye platform. Negotiating compromises is also essential when it comes to tasks that are not considered as research by either party, for example, software engineering for computer science researchers or performing manual annotations for (digital) humanities scholars, even though both tasks are necessary for progress in each field. When stepping into any interdisciplinary academic collaboration, we believe that it is crucial to be aware of and to clearly define the level of expectation and commitment given to different tasks.

## 6 CONCLUSION

In the NewsEye project, we learned that three aspects have been hindering successful interdisciplinary research on historical newspapers: (a) different motivations, goals, needs, and assumptions when developing/using digital tools, (b) a lack of understanding of the differences between disciplines, and (c) tasks that are necessary for collaborative research but not scientifically interesting for any party.

Based on literature and our experiences in the NewsEye project, we suggest that a workflow‐oriented view can help avoid some of the issues. Workflows are a convenient tool for discussing various concepts, processes and practices between the involved scholars. They can help make implicit assumptions explicit (point 1 above), increase understanding between disciplines (point 2) and also identify and motivate tasks that otherwise could be neglected (point 3).

We proposed a more specific integrated hermeneutics workflow, which merges process‐oriented approaches and critical reflections in the sense of digital hermeneutics. In this workflow, we emphasized a close interaction between the analytical, exploratory, interpretative, and contextualizing steps, underlined the importance of qualitative research steps throughout the workflow and highlighted critical reflection as an essential part of the workflow that starts with the digitization and organization and ends with the dissemination.

We gave three examples of digital hermeneutics in action, explaining how user interfaces and tools can support critical reflection in heuristic, in (source) criticism, and in interpretation. We presented new methods and developments for research with digital newspapers. We showed that successful interdisciplinary collaboration and research needs more than shared visions and goals in order to find common approaches that support equally the development of historical research questions, the creation of transparent digital methods, and the interpretation of analysis and search results.

The presented integrated digital hermeneutics workflow serves both as a model for structuring research into historical newspapers and as a conceptual tool for discussing interdisciplinary expectations and commitments when planning interdisciplinary research.

## Endnotes

^1^

https://www.europeana.eu/de/collections/topic/18-newspapers

^2^
A digital investigator for historical newspapers: https://www.newseye.eu

^3^
Mapping networks of reprinting in 19th‐century newspapers and magazines: https://viraltexts.org/

^4^
Tracing global information networks in historical newspaper repositories from 1840 to 1914: https://oceanicexchanges.org/

^5^
Mining 200 years of historical newspapers: https://impresso-project.ch/

^6^
A platform developed by Teklia for the automatic processing of large collections (document management, manual annotation, automatic transcription, and named entity extraction) of scanned or handwritten documents with focus on historical document analysis and understanding, https://teklia.com/research/projects/

^7^
A project that will take a fresh look at the well‐known history of the industrial revolution using data‐driven approaches: https://www.turing.ac.uk/research/research-projects/living-machines

^8^
A short version of the humanities’ experience has been published in issue 16 of the EuropeanaTech Insight: https://pro.europeana.eu/page/issue-16-newspapers#doing-historical-research-with-digital-newspapers

^9^

https://readcoop.eu/transkribus/?sc=Transkribus

^10^

https://platform.newseye.eu/

^11^

https://impresso-project.ch/

^12^

https://www.youtube.com/watch?v=N85F1JXdfS4

^13^

https://glam-workbench.net/trove-newspapers/#save-a-trove-newspaper-article-as-an-image

^14^

http://data.cervantesvirtual.com/blog/labs/

^15^

https://github.com/NewsEye/NLP-Notebooks-Newspaper-Collections
